# Whole genome microarray analysis, from neonatal blood cards

**DOI:** 10.1186/1471-2156-10-38

**Published:** 2009-07-22

**Authors:** Jill Hardin, Richard H Finnell, David Wong, Michael E Hogan, Joy Horovitz, Jenny Shu, Gary M Shaw

**Affiliations:** 1 University of California Berkeley, School of Public Health, Berkeley, CA, 94720, USA; 2 Texas A&M, Institute of Biosciences and Technology, Houston TX, 77030, USA; 3 March of Dimes, California Research Division, Children's Hospital Oakland Research Institute, Oakland, CA, 94609, USA; 4 GenVault Corporation, Carlsbad CA, 92011, USA; 5 Expression Analysis Corporation, Durham, NC, 27713, USA

## Abstract

**Background:**

Neonatal blood, obtained from a heel stick and stored dry on paper cards, has been the standard for birth defects screening for 50 years. Such dried blood samples are used, primarily, for analysis of small-molecule analytes. More recently, the DNA complement of such dried blood cards has been used for targeted genetic testing, such as for single nucleotide polymorphism in cystic fibrosis. Expansion of such testing to include polygenic traits, and perhaps whole genome scanning, has been discussed as a formal possibility. However, until now the amount of DNA that might be obtained from such dried blood cards has been limiting, due to inefficient DNA recovery technology.

**Results:**

A new technology is employed for efficient DNA release from a standard neonatal blood card. Using standard Guthrie cards, stored an average of ten years post-collection, about 1/40^th ^of the air-dried neonatal blood specimen (two 3 mm punches) was processed to obtain DNA that was sufficient in mass and quality for direct use in microarray-based whole genome scanning. Using that same DNA release technology, it is also shown that approximately 1/250^th ^of the original purified DNA (about 1 ng) could be subjected to whole genome amplification, thus yielding an additional microgram of amplified DNA product. That amplified DNA product was then used in microarray analysis and yielded statistical concordance of 99% or greater to the primary, unamplified DNA sample.

**Conclusion:**

Together, these data suggest that DNA obtained from less than 10% of a standard neonatal blood specimen, stored dry for several years on a Guthrie card, can support a program of genome-wide neonatal genetic testing.

## Background

Dried neonatal blood, stored and processed on filter paper, has been the standard for neonatal screening for 50 years [1]. The ordinary use of neonatal blood is based upon the excision of blood spot punches, typically 3 mm-6 mm in diameter, followed by physical or biochemical analysis of serum analytes released from the punch by soaking in alcohol or water [2]. More recently, dried blood spots have been used to screen for heritable traits at the DNA level, typically traits such as cystic fibrosis and the thalassemias, and other traits that are readily assayed by PCR tests [3,4].

In 2005, based on recent advances made in highly parallel microarray technology, geneticists at March of Dimes proposed that we may be entering an era where the DNA complement of such dried blood spots might be sufficient, in terms of quantity and quality, to support genome-wide analysis of complex heritable traits, thereby leapfrogging the limits of single-gene analysis[5].

In spite of the exciting prospects implied by that 2005 review, relatively little work has been published in the intervening three years to validate such genome-scale neonatal screening [6,7]. Microarray technology has improved significantly in that period, in terms of diminished cost and sample requirement, and has yielded increased data density and quality [8]. However, such genome-scale microarray analysis continues to require an input DNA mass (about 250 ng) that is about 100 times larger than required for simple PCR testing; requires DNA that is double stranded; and requires DNA with a length-span that is about 5 times longer than required for most PCR reactions. Thus, going forward, it is suggested here that a major technical barrier to the adoption of genome-wide microarray technology may not be the microarray technology per se, but instead, may be the quantity and quality of DNA that can be usefully recovered from an ordinary air-dried neonatal blood specimen.

The importance of DNA recovery from such Guthrie cards is discussed at length in a recent comparative study by Sjoholm and colleagues [9]. They have compared a number of commercially available kits and procedures for DNA recovery from Guthrie cards and have show that only about 15%–25% of the total DNA complement can be recovered. They have measured DNA recovery from dried blood spots stored for up to 26 years, and have shown that, on standard 3 mm punches from such cards, DNA yields (with the best available technology) are only about 30 ng per punch.

However, in spite of the relatively low yields, Sjoholm have shown that the small amount of DNA obtained remains an excellent substrate for whole genome amplification, and relatively complex multiplex SNP analysis [9]. However, for genome wide scanning methods such as microarray analysis (which require at least 250 ng of input DNA) the relatively low DNA recoveries, obtained by Sjoholm, would require extraction and pooling of as many as eight 3 mm punches: a value that is difficult to reconcile for such rare specimens.

Generally similar results have been obtained by Mas (10) in a study of dried blood spots stored on treated filter paper matrices such as Whatman FTA or IsoCode, employing the manufacturer's extraction method. In that study, about 25% recovery was obtained in a single extraction, to yield up to 150 ng of single stranded DNA as a 200 uL solution, per 40 uL of adult human blood input [10]. As for the by Sjoholm, the DNA obtained by Mas et. al. could be used effectively for multiplex PCR and for whole genome amplification, but as the authors correctly noted, might be too dilute too support more complex studies such as genome wide microarray analysis. Moreover, since the DNA extraction procedures employed by Mas yielded denatured DNA, the product of such extractions would not be applicable to methods such as Affymetrix microarrays, which require that the DNA substrate remains in a native, double stranded state prior to analysis.

Here, we describe the use of a new technology, referred to as GenSolve™, originally developed as a high-efficiency method to recover native DNA from blood spots on chemically treated FTA filter paper [11] but used here to recover DNA from neonatal blood spots on standard Guthrie cards collected from 1991 to 2003 as part of the California Birth Defects Monitoring Program. The GenSolve technology was used in combination with standard DNA purification, followed by analysis on the Illumina 610 bead array microarray platform, which interrogates about 610,000 sites of human SNP variation in parallel [12].

Although the Illumina 610 chip does not contain content that was developed specifically for neonatal screening, the scale of the analysis performed on the Illumina 610 chip can be viewed as a technical surrogate for any large panel of genome-wide SNP testing that could be developed as a screening tool. For the immediate future, neonatal screening will likely continue to employ biochemical analysis mediated by tandem mass spectrometry and related methods, to which genetic testing will be added, in parallel. Typically, only a small fraction of a dried neonatal sample will be available for microarray or microarray-like genetic analysis. Thus, the work described here is focused on microarray testing using DNA obtained from only two 3 mm diameter Guthrie card punches, roughly 1/40^th ^of the blood ordinarily collected from a neonate, on a standard 5-spot Whatman 903 Guthrie card [13].

## Results and discussion

### DNA recovery

Table 1 presents the purified DNA recovery from 24 dried neonatal blood samples obtained from the California Birth Defects Monitoring Program. The average pooled DNA recovery from two identical 3 mm punches, via the GenSolve technology, was measured to be 260 ng +/- 70 ng. Based on the yields obtained by Sjoholm (9) the data presented in Table 1 suggest that the observed DNA recovery from paired 3 mm punches (260 ng) obtained with GenSolve is approximately 4 times greater than would be predicted from previously studied methods (60 ng). Assuming 0.6 uL of dried blood wetting per mm^2 ^[14] the average yield per two such 3 mm diameter punches, distributed over 14 mm^2^, corresponds to an average DNA recovery of 19 ng/mm^2^, averaged over the full 24 sample set, and an average DNA recovery of 30 ng/uL relative to the original fluid blood specimen, which is near to the value expected for 100% DNA recovery.

**Table 1:** DNA Recovery from Two 3 mm Guthrie Card Punches

sample ID	Yield (ng)	Conc (ng/uL)	Specimen Name	Collection Date
1	261.7	26	530011	1995
2	203.37	20	529979	1995
3	162.33	16	529969	2003
4	312.9	31	529943	2003
5	380.43	38	529844	1994
6	212.16	21	529895	1994
7	319.9	32	520908	2001
8	188.41	19	529809	2001
***9***	***246.88***	***65***	***MOD881***	***1993***
***10***	***244.55***	***65***	***MOD892***	***1993***
11	225.47	23	529874	2003
12	131.31	13	529810	2003
***13***	***391.96***	***50***	***MOD919***	***1998***
***14***	***367.67***	***48***	***MOD937***	***1998***
15	161.23	16	529852	2000
16	264.59	26	529906	2000
***17***	***255.67***	***57***	***MOD920***	***1995***
***18***	***226.43***	***65***	***MOD975***	***1995***
***19***	***268.02***	***69***	***MOD986***	***1995***
***20***	***234.94***	***53***	***MOD001***	***1995***
21	350.78	35	530127	1995
22	339.53	34	530128	1991
23	224.1	22	529236	1992
24	274.61	27	529237	1997
***overall average***	***<260 ng>***	***<26 ng/uL>***		***<1997>***
***Microarray samples***	***<280 ng>***	***<59 ng/uL>***		***<1995>***

DNA concentration was measured via PicoGreen analysis. Samples in red refer to those specimens used for microarray analysis, based on the requirement that their DNA concentration exceed 50 ng/uL to accommodate Illumina hybridization chemistry. Notice that the average DNA yield (in nanograms) for the 8 specimens used for microarray analysis and their average age did not deviate significantly from the average of all 24. However, due to volume variability in the final concentrated DNA sample, the mass concentration of the 8 specimens used for microarray analysis (59 ng/uL) was approximately twice that of the average over the entire set of twentyfour (26 ng/uL).

### DNA Quality

**Figure 1:**
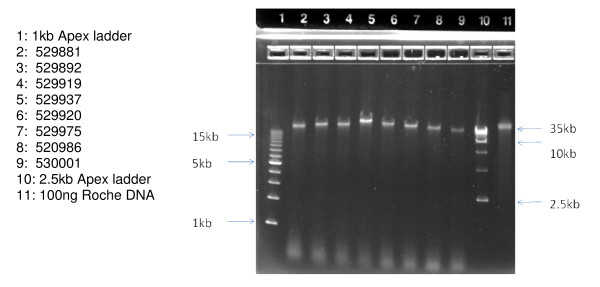
**Samples to be used for Illumina 610 Microarrays, *without prior WGA***. Samples were applied at 100 ng per well and run on an Invitrogen 0.8% agarose E-gel for 30 minutes, and visualized by ethidium bromide staining.

**Figure 2:**
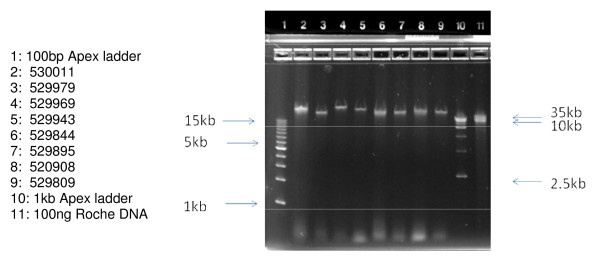
**Samples *not used *for Illumina 610 Microarrays, *without prior WGA***. Samples were applied at 100 ng per well and run on an Invitrogen 0.8% agarose E-gel for 30 minutes, and visualized by ethidium bromide staining.

**Figure 3:**
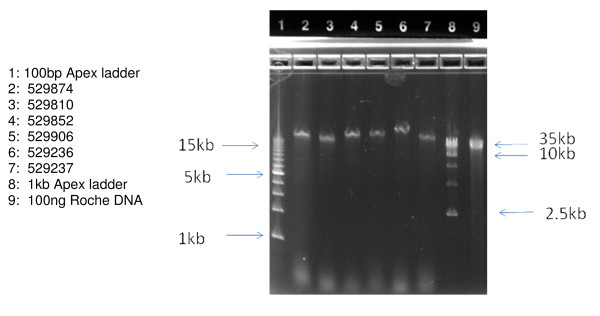
**Samples *not used *for Illumina 610 Microarrays, *without prior WGA***. Samples were applied at 100 ng per well and run on an Invitrogen 0.8% agarose E-gel for 30 minutes, and visualized by ethidium bromide staining.

The length of the DNA obtained was estimated using 0.8% agarose gel electrophoresis. As seen in Figure 1, 100 ng of each sample chosen for microarray analysis (lanes 2–9) was compared to 100 ng of very high molecular weight Roche DNA control (lane 11). In all cases, the Guthrie card DNA samples are present as a single collapsed band which migrates in the 40 kb range, relative to external size standards (lanes 1 & 10). Similar analysis has been performed on the remaining 15 samples, not used for microarray analysis (Figures 2 &3). Taken together these data indicate that the majority of the DNA isolated from these Guthrie cards is longer than 40 kb.

### Whole Genome Amplification

**Figure 4:**
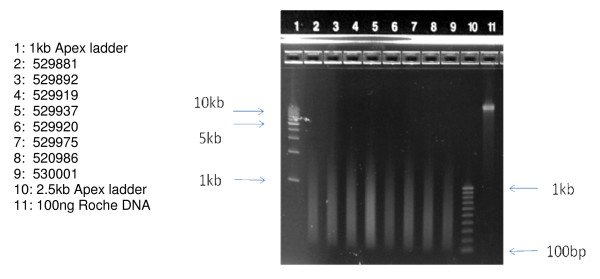
**Samples to be used for Illumina 610 Microarrays, *subsequent to WGA***. Samples were applied at 100 ng per well and run on an Invitrogen 1.2% agarose E-gel for 30 minutes, and visualized by ethidium bromide staining.

To determine if the DNA extracted from these Guthrie card samples might be extended to a larger number of DNA tests, PCR-based whole genome amplification was performed on the 8 samples to be analyzed on the Illumina 610 microarray platform. The Sigma-Rubicon amplification technology was employed [15] which, as a necessary first-step in the process, induces chemical shearing of the DNA template to about 200 bp-1000 bp, which is followed by thermal cycling to affect a 100–1000 fold mass amplification. The yield of those reactions is summarized in Table 2. Beginning with 1 ng of Guthrie card DNA, a final product yield of 1 ug-2 ug is obtained in all cases. Since only about 1/250^th ^of the original purified DNA sample was used in these reactions, the data of Table 2 illustrate that, when coupled to Sigma-Rubicon WGA, the DNA content of two 3 mm dried blood spots could (with pooling) be amplified to a final yield of greater than 200 ug, thus enabling a broader range of applied genetics applications. As seen in Figure 4, 100 ng of the amplified product is characterized by the expected size distribution range from 200 bp to about 1,000 bp on a 1.4% agarose gel. It is interesting to note that this Guthrie card DNA, used at 1 ng, produced, as expected, roughly 5–10 fold less amplified product than a highly purified DNA reference sample at 10 ng of template input, suggesting that the Guthrie card DNA behaved as a similar amplification substrate.

**Table 2:** Whole Genome Amplification, from 1 ng of DNA, Recovered from Two 3 mm Guthrie Card Punches

Sample ID	Specimen Name (1 ng input)	260	280	260/280	ug/ul	Total (ug)
9	WGA 881	0.04	0.03	1.78	0.03	1.19
10	WGA 892	0.05	0.03	1.68	0.03	1.38
13	WGA 919	0.06	0.03	1.94	0.04	1.67
14	WGA 937	0.08	0.05	1.79	0.05	2.27
17	WGA 920	0.04	0.03	1.79	0.03	1.19
18	WGA 975	0.06	0.04	1.75	0.04	1.67
19	WGA 986	0.06	0.04	1.68	0.04	1.73
20	WGA 001	0.05	0.03	1.65	0.03	1.46
	Human gDNA CTL (10 ng input)	0.39	0.2	1.96	0.23	10.4
	Blank	0.01	0.01	1.43	0.01	0.3

DNA concentration of the amplified product was measured by NanoDrop Analysis

### Illumina 610 Microarray Analysis

Eight primary DNA isolates (underlined in Table 1) and the corresponding whole genome amplified products of those eight (Table 2) were used for microarray analysis on the Illumina 610 microarray platform. Although the overall DNA yield from these eight samples (280 ng) was only marginally higher than the average over the entire set of 24 (260 ng) the eight which were chosen for microarray analysis all presented with a mass concentration greater than 50 ng/uL which is the minimum required for the Illumina platform. That variability in concentration, but not total yield, is due to the difficulty in controlling the volume obtained during the final Microcon concentration step. It should be noted that the average collection date of the eight specimens chosen (1995) is not significantly different than the average of the full set of 24 (1997).

The quality of those DNA samples as a substrate for microarray analysis has been summarized as the SNP call rate, which is a measure of the fraction of 610,000 SNP assays performed by the array. It is generally accepted that SNP call rates in excess of 90% are acceptable, while those in excess of 94% are considered to be high quality, and those in excess of 99% are considered to be very high quality [16]. As seen in Table 3, all eight of the primary DNA isolates gave SNP call rates greater than 99%. Microarray data quality derived from 1 ng of the identical sample, but whole genome amplified prior to microarray analysis, showed uniform diminishment of microarray SNP call rate to 94% or greater, which may be attributable to the (expected) reduction of template length incurred during amplification (see Figure 4).

**Table 3:** Illumina 610 Microarray Data: QC Analysis On DNA Obtained from Two 3 mm Guthrie Card Punches

**Specimen Name**	**No Calls**	**Calls**	**Total**	***Call Rate (%)***
**Primary DNA Isolate**				
MOD 881	1484	591008	592492	***99.75***
MOD 892	1581	590911	592492	***99.73***
MOD 919	1502	590990	592492	***99.75***
MOD 937	1607	590885	592492	***99.73***
MOD 920	2524	589968	592492	***99.57***
MOD 975	2840	589652	592492	***99.52***
MOD 986	2432	590060	592492	***99.59***
MOD 001	3240	589252	592492	***99.45***
**Corresponding Whole Genome Amplified DNA**				
WGA 881	31699	560793	592492	***94.65***
WGA 892	19207	573285	592492	***96.76***
WGA 919	27928	564564	592492	***95.29***
WGA 937	17094	575398	592492	***97.11***
WGA 920	27680	564812	592492	***95.33***
WGA 975	27080	565412	592492	***95.43***
WGA 986	22501	569991	592492	***96.20***
WGA 001	34458	558034	592492	***94.18***

SNP call rates were determined by Illlumina BeadArray analysis.

An alternative, more rigorous assessment of quality for those samples was next obtained on the Illumina 610 microarray platform. Microarray data from unamplified and the corresponding WGA-amplified samples were compared, pair-wise, using Illumina statistical software [17]. These data are presented in Table 4, and indicate that among the approximately 600,000 SNP loci that were measured in both sample types, concordance between measured data was in excess of 99%, thus illustrating that the WGA-amplified material presents an accurate reflection of genetic variation in the sample tested.

**Table 4:** Primary DNA vs Whole Genome Amplified DNA Analyzed on Illumina 610 Microarrays

***Sample1 Primary Isolate***	***Sample2 Whole Genome Amplified***	**No Call in one of the samples**	**No Call in Both Samples**	**Concordance**	**Discordance**	**Concordance Rate (%)**
MOD 881	WGA 881	31221	981	559134	1156	99.79
MOD 892	WGA 892	18592	1098	572300	502	99.91
MOD 919	WGA 919	27450	990	563096	956	99.83
MOD 937	WGA 937	16551	1075	574418	448	99.92
MOD 920	WGA 920	27354	1425	562951	762	99.86
MOD 975	WGA 975	26404	1758	563599	731	99.87
MOD 986	WGA 986	22113	1410	568375	594	99.9
MOD 001	WGA 001	33998	1850	555479	1165	99.79

Concordance between sample pairs was determined by Illumina BeadArray analysis.

## Conclusion

We have demonstrated that high quality DNA can be obtained from a standard neonatal blood screening card, stored dry for at least 10 years. In at least 1/2 of the samples tested, about 1/40^th ^of a standard dried blood sample on Guthrie card (two 3 mm punches) is sufficient for genome wide microarray analysis. The data also suggest that, when that primary DNA is amplified via the Sigma-Rubicon method, as little as 1 ng of the recovered DNA can be used for genome wide microarray analysis. Thus, these data suggest that, for other methods of genome-wide scanning that require multiple-microgram quantities of DNA, only a few nanograms of the primary DNA isolate might be similarly amplified and pooled for use.

The total DNA yields were at or above the useful microarray range (250 ng) for about 2/3 of the Guthrie card specimens tested, whereas the remaining 1/3 of the samples yielded DNA in the range from 130 ng to 250 ng. Thus, the data illustrate that the larger surface area, and hence greater amount of dried blood specimen obtained from three 3 mm punches or a single 6 mm punch might be a more dependable sample source, and might produce enough DNA from microarray analysis from most neonatal specimens. Since 6 mm Guthrie card punching is routine in the current practice of automated, high throughput neonatal screening, the data here suggest that a standard 6 mm Guthrie card punch could (as first envisioned in 2005) might become the basis for genome-wide neonatal testing. Confirmation of that prediction is ongoing.

## Methods

### Neonatal Blood Specimens

A series of standard dried neonatal blood specimens were obtained on standard Whatman 903 "Guthrie" cards (GE-Whatman Corporation) over the period from 1991 to 2003 by the California Birth Defects Monitoring Program as part of a larger genetic investigation of risk factors of birth defects. Use of the human specimens for the purposes of the main study and the current sub-study was approved by the California Committee for the Protection of Human Subjects. Duplicate aliquots were excised for analysis by hole-punching with a standard 3 mm Harris punch. Both were then transferred to a 1.5 ml microfuge tube for subsequent processing.

### DNA Extraction from Neonatal Blood Cards

DNA is a large polymer chain compared to the size of the pores of the Guthrie card filter paper, thus restricting DNA release from dried blood upon rehydration. The traditional methods to facilitate DNA release from Guthrie card filter paper require denaturation of the DNA using a strong alkaline compound or by heating (10) thus compromising the physical and chemical integrity of the stored DNA and rendering the DNA unusable for microarray analysis, which requires that DNA be recovered in its native, double stranded form. Here we have employed a simple technology, "GenSolve™", that allows genomic DNA in its native, double stranded form to be released from dried blood on filter paper, concurrent with ordinary protease treatment of the blood. The manufacturer's standard protocol was employed as described for processing of treated FTA paper (GenVault Corporation). Briefly, two 3 mm punches from a Guthrie card were pooled and incubated for one hour at 65C, with shaking, in a standard 1.5 ml microfuge tube, in the presence of 400 ml of GenSolve Solution A, 100 uL of Savinase solution, in 1% LiDS, overall.

Subsequent to inactivation with GenSolve Solution B, the resulting solution phase was isolated by centrifugation through a spin basket, then loaded directly onto a Qiagen QiaAmp Mini column for DNA purification (Qiagen Corporation). The resulting DNA eluate was concentrated to 5–10 uL on a Microcon Y100 membrane (Millipore Corporation), and then transferred to a 0.6 ml microfuge tube for storage at 4C until use in Illumina 610 microarray analysis. Since a final volume in the 5 uL–10 uL range is difficult to standardize on a Microcon filter, we have observed that the final DNA concentrations obtained (as in Table 1) were more variable than the total DNA yields, due to variability in the final sample volume.

### DNA Quality Analysis

The purified DNA complement from Guthrie cards was quantified by PicoGreen analysis (Invitrogen Corporation) relative to both external and internal standards and recorded as both total yield (nanograms) and DNA mass concentration (ng/uL). PicoGreen is specific for double stranded DNA and provides an accurate measure of double stranded DNA content. DNA fragment length was measured by electrophoresis using 100 ng of DNA on pre-cast 0.8% agarose gels (E-gel, Invitrogen) or for shorter, amplified DNA samples, on 1.2% agarose gels. In all instances 100 ng of each purified sample was loaded per gel lane (based on PicoGreen quantitation) and compared to an identical amount of a RocheGen DNA standard, with a known mass in the 100 kb–200 kb range. On a 0.8% agarose gel assay, DNA fragments with a length greater than about 40 kb will migrate as a single collapsed band, which relative to a high molecular weight standard, can be used to estimate the fraction of the unknown sample greater than about 40 kb. On a 1.2% agarose gel, DNA fragments with a length greater than about 10 kb will migrate as a single collapsed band, which is an estimate of that fraction of the sample greater than 10 kb.

### Whole Genome Amplification

Roughly 50% of the 5 uL–10 uL samples obtained from Guthrie cards were concentrated enough for use in microarray analysis. 1 ng (about 0.5% of the purified DNA sample) from each sample, was subjected to whole genome amplification using the Sigma-Rubicon PCR based technology, per the manufacturer's protocol (Sigma-Aldrich Corporation) under CLIA control at Expression Analysis (Raleigh-Durham NC). The resulting whole genome amplified product was then diluted to 50 ng/uL and used for Illumina 610 microarray analysis.

### Illumina 610 Microarray Analysis

Eight specimens out of the full set of 24 were chosen for microarray analysis (Table 1, underlined). The total DNA yield (Table 1, second column) and average age of the 8 chosen (Table 1, last column) did not deviate significantly from the average of all 24. However, due to volume variability in the final concentrated DNA sample, the average mass concentration of the 8 specimens chosen for microarray analysis (59 ng/uL) was approximately twice that of the average over the entire set of twentyfour (26 ng/uL) thereby matching or exceeding the DNA mass concentration (50 ng/uL) required for optimal Illumina 610 microarray performance: see for instance ref 16.

Two hundred nanograms of each sample were then analyzed by the microarray laboratories of Expression Analysis (Raleigh-Durham NC). Briefly, all samples were analyzed on the Illumina 610M microarray platform and subjected to preliminary statistical analysis to generate a SNP call rate, which is a standard metric used to assess data quality. Both unamplified and Sigma-Rubicon whole genome amplified samples were measured on the Illumina 610 microarray platform and then additionally subjected to pair-wise concordance analysis using standard methods.

## Authors' information

RHF is Professor and Director of the Texas A&M Institute of Biosciences & Technology and a specialist in neonatal screening. MH is Chief Scientific Officer of GenVault Corporation, inventor and developer of technologies for biobanking and dry state specimen preservation and recovery. GMS is Research Director of the California March of Dimes, and previously, Research Director/Senior Epidemiologist California Birth Defects Monitoring Program

## Authors' contributions

JH conceived of the study and acquired samples. RHF co-directed the study. DW prepared samples for analysis. MH conceived of and co-directed the study. JH and JS performed microarray analysis. GMS co-directed the study. All authors read and approved the final manuscript.
